# Supplementary material for: A DARPin promotes faster onset of botulinum neurotoxin A1 action
Source: Nat Commun. 2023 Dec 18;14:8317. doi: 10.1038/s41467-023-44102-4 (PMC10728214; doi:10.1038/s41467-023-44102-4)
Supplement: Supplementary file 1 — Supplementary Information [file 41467_2023_44102_MOESM1_ESM.pdf]

A DARPin Promotes Faster Onset of Botulinum Neurotoxin A1 Action

Oneda Leka[1]*, Yufan Wu[1]*, Giulia Zanetti[2], Sven Furler[3], Thomas Reinberg[3], Joana Marinho[3], Jonas Schaefer[3], Andreas Plückthun[3], Xiaodan Li[1], Marco Pirazzini[2] and Richard A. Kammerer[1]

[1] Laboratory of Biomolecular Research, Division of Biology, Paul Scherrer Institut, 5232 Villigen PSI, Switzerland.

[2] Department of Biomedical Sciences, University of Padova, 35121 Padova, Italy.

[3] Department of Biochemistry, University of Zurich, 8057 Zurich, Switzerland.

* These authors contributed equally to the study.

Corresponding Author:

Richard A. Kammerer, Laboratory of Biomolecular Research, Division of Biology, Paul Scherrer Institut, CH-5232 Villigen PSI, Switzerland.

8xHis-tagged DARPin-F5	MRGSHHHHHHHHGSDLGKKLLEAARAGQDDEVRILMANGADVNAVDMHG<br>YTPLHLAAAVGHLEIVEVLLKAGADVNAWDQVGKTPLHLAAKWGHLEIVEVLL<br>KHGADVNAQDWMGRTPFDLAIDNGNEDIAEVLQKAAKLNDYKDDDDK
Truncated DARPin-F5 with cleavable 8xHis tag	MRGSHHHHHHHHGSGLVPRDLGKKLLEAARAGQDDEVRILMANGADVNAV<br>DMHGYTPLHLAAAVGHLEIVEVLLKAGADVNAWDQVGKTPLHLAAKWGHLE<br>IVEVLLKHGADVNAQDWMGRTPFDLAIDNGNEDIAEVLQKAAKLN~~DYKDDD~~<br>~~DK~~<br><br>LVPR GS- Thrombin cleavage site

Supplementary Figure S1. Amino-acid sequence of DARPin-F5 and its truncated version with a cleavable 8xHis tag.

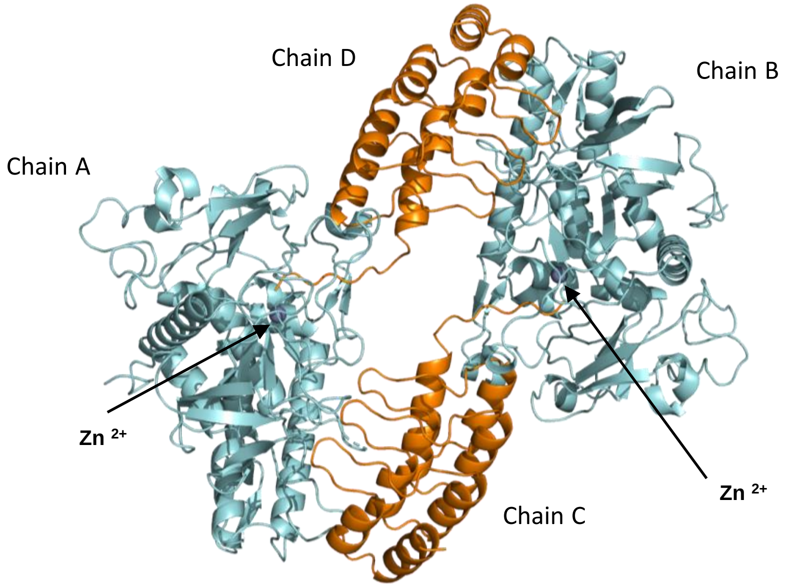

Supplementary Figure S2. Crystal structure of the LC/A1-DARPin-F5 complex. Cartoon representation of the four polypeptide chains in the asymmetric unit. (PDB code 8HKH). LC/A1 is shown in cyan (chain A and B) and DARPin-F5 (chain C and D) in orange. The zinc ion is shown as grey sphere.

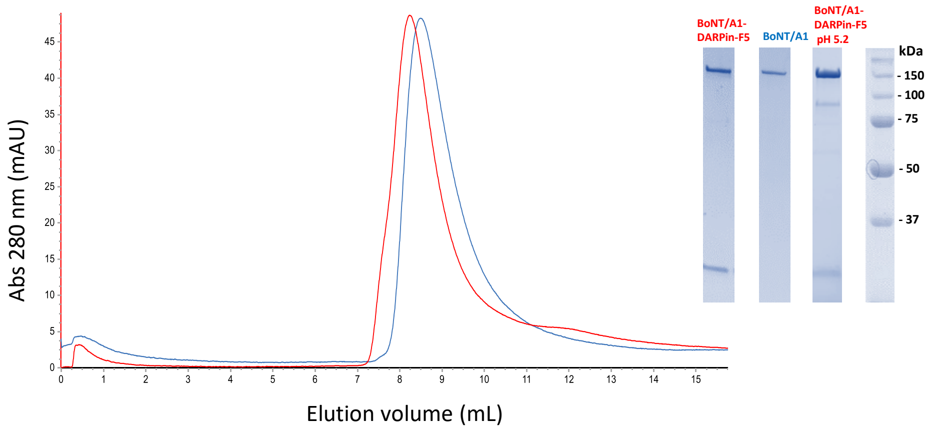

Supplementary Figure S3. Size exclusion chromatography (SEC) and SDS-PAGE analysis of BoNT/A1-DARPin-F5 complex. BoNT/A1 (blue trace), BoNT/A1-DARPin-F5 (red trace). SEC analysis was performed with a Superdex 75 10/300 column (GE Healthcare) in 20 mM Tris-HCl pH 7.4, 100 mM NaCl (or in 20 mM Sodium Acetate pH 5.2, 100 mM NaCl) and fractions from respective peaks were analyzed on a gradient SDS-PAGE gel (BioRad). Left side gel fraction from peak complex BoNT/A1-DARPin-F5 at pH 7.4, middle gel BoNT/A1 alone (153 kDa), right side gel fraction from peak complex BoNT/A1-DARPin-F5 at pH 5.2. The experiments were repeated twice independently and yielded similar results.

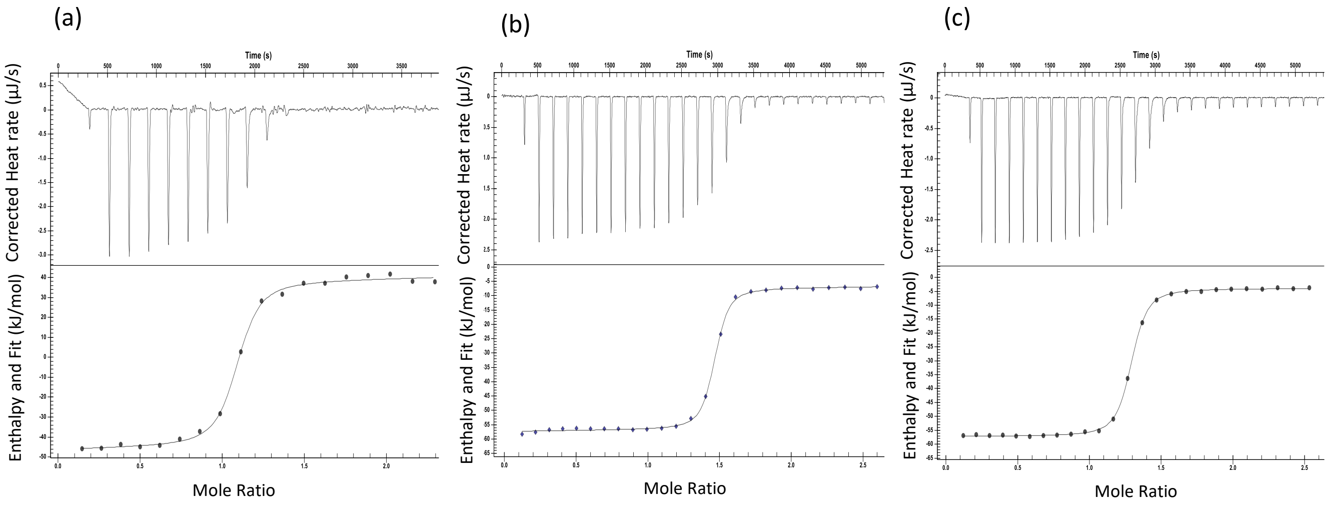

Supplementary Figure S4. ITC titrations of DARPin-F5 variants to LC/A1. Representative ITC titrations of DARPin-F5 to LC/A1 at pH 7.5 with (a) and without (b) the N-terminal 8xHis tag on DARPin-F5 and (c) without the N-terminal 8xHis tag on DARPin-F5 at pH 5.5. (a) $K_D = 9{,}61 \times 10^{-8} \pm 0.02$ M, (b) $K_D = 4{,}43 \times 10^{-8} \pm 0.01$ M, (c) $K_D = 7{,}01 \times 10^{-8} \pm 0.01$ M. The error is the standard deviation on at least two independent measurements.

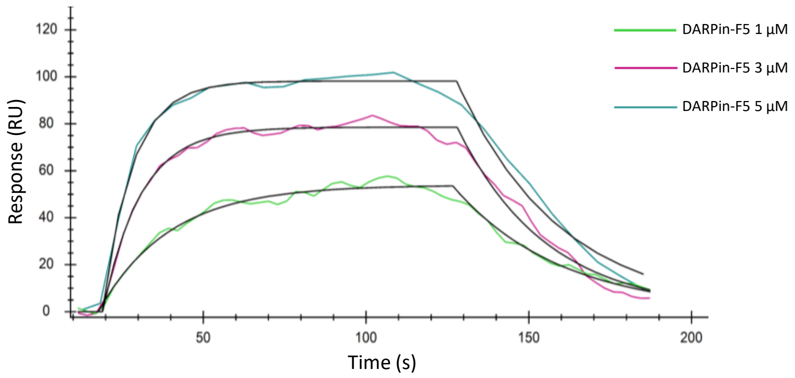

Supplementary Figure S5. Binding affinity and kinetic parameters of the interaction of DARPin-F5 with LC/A3.

SPR analysis of DARPin-F5 binding to the LC/A3, performed on the Nicoya OpenSPR™; overlaid with a fit of 1:1 binding model (black line). Increasing DARPin-F5 concentrations ranging from 1 to 5 µM were applied. The K_D value of 1.68 ± 0.58 µM was calculated from the raw data using the TraceDrawer Software (Ridgeview Instruments AB).

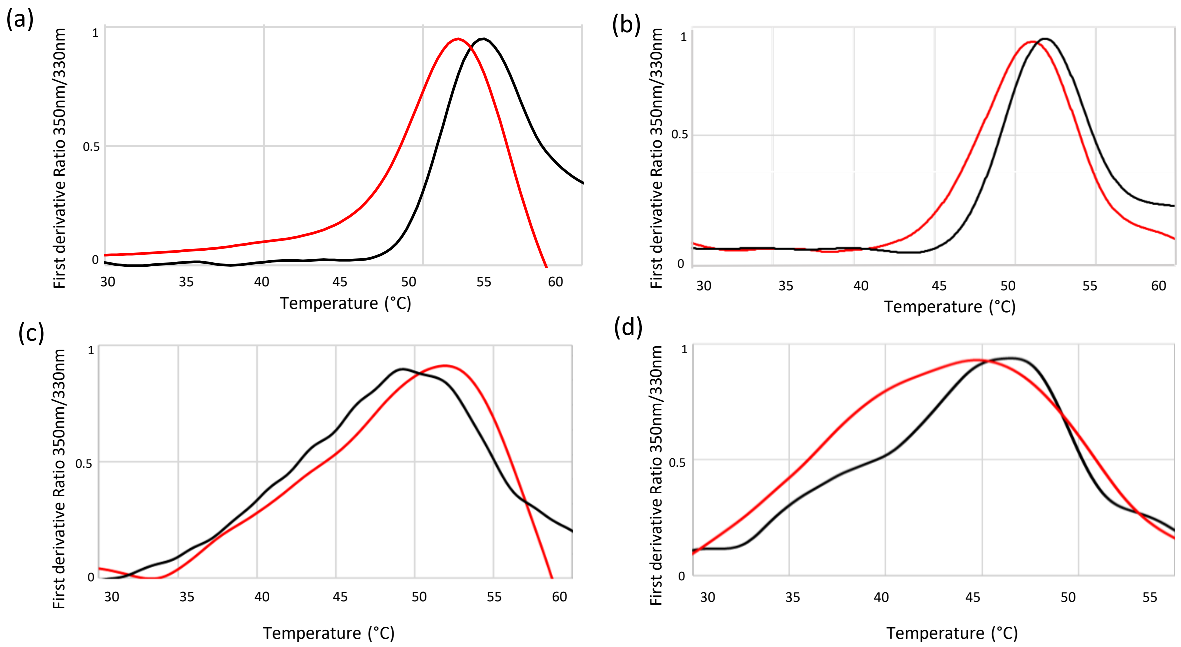

Supplementary Figure S6. Thermal stability of the BoNT/A1-DARPin-F5 and LC/A1-DARPin-F5 complexes. First derivatives of the fluorescence ratio (350nm/330nm) are shown as a function of temperature for BoNT/A1-DARPin-F5 (red) and for BoNT/A1 alone (black) at (a) pH 5.5 and (b) pH 7.4. (c) and (d) Same experiment shown for LC/A1-DARPin-F5 (red) and for LC/A1 alone (black) at pH 5.5 and pH 7.4. Each curve is the average of three independent measurements.

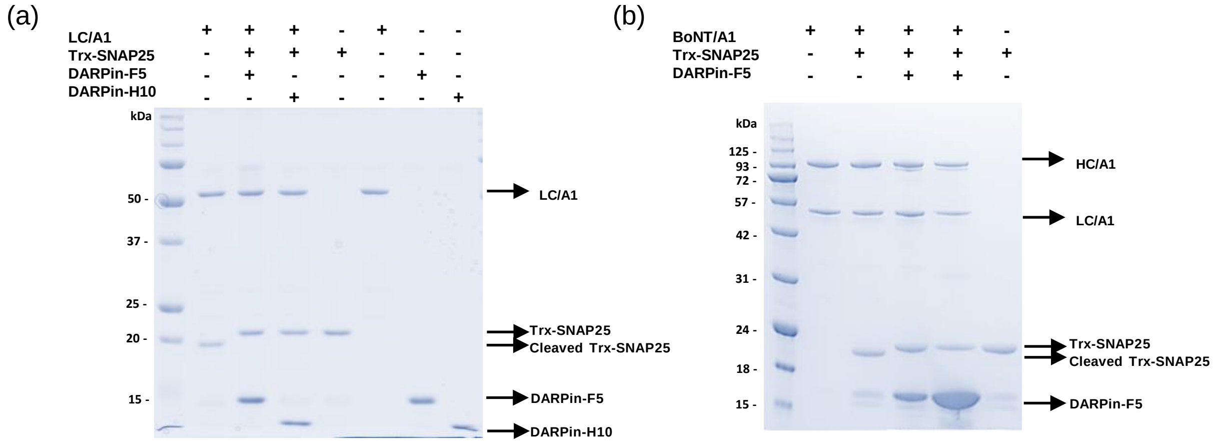

Supplementary Figure S7. Uncropped gels of data shown in figure 1a and b. The experiments were repeated twice independently and yielded similar results.

Figure 3c

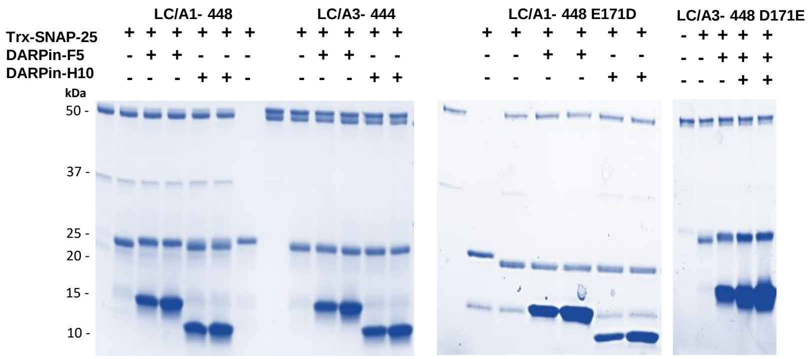

Figure 5b

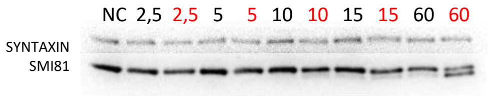

Supplementary Figure S8. Above, uncropped gels of data shown in figure 3c. Below, supplementary western blot of data shown in figure 5b. In both cases, experiments were repeated twice independently and yielded similar results.

Data collection	
Resolution range (Å)	49.19 - 2.55 (2.62 - 2.55)
Space group	*P*1
Polypeptide chains/AU	4
Unit cell parameters	
a, b, c (Å)	59.56, 59.63, 102.18
α, β, γ (°)	74.976, 89.904, 73.154
Observed reflections	147652 (10839)
Unique reflections	39460 (2962)
Multiplicity	3.7 (3.8)
Completeness (%)	93.4 (94.2)
Mean I/sigma(I)	4.49 (1.17)
CC(1/2)	0.976 (0.452)
Wilson B-factor	47.03
Refinement	
Resolution range (Å)	49.19 - 2.7 (2.797 - 2.7)
R_{work} (%)	26.17
R_{free} (%)	30.61
Protein atoms	8840
rmsd of bond lengths	0.003
rmsd of bond angles	0.558
Average *B*-factor (Å^2)	
Total Protein Complex	47.75
LC/A1 (chain A/B)	47.80
DARPin-F5 (chain C/D)	51.71
Ligands	42.38
Solvent	31.97
Ramachandran plot (%)	
Favored	97.45
Allowed	2.55
Outliers	0
Crystallization condition	0.1 M HEPES pH 7.5<br>28 % w/v Jeffamine ED-2003
Values in parentheses refer to the outermost resolution shell.	

Supplementary Table S1. Values in parentheses refer to the outermost resolution shell.

Residue (LC/A1 chain A)	Distance (Å)	Residue (DARPin-F5 chain C)
Glu 171 [O]	3.5	Arg 25 [NE]
Glu 171 [OE1]	3.2	Tyr 50 [OH]
Glu 171 [OE2]	2.5	Lys 91 [NZ]
Lys 128 [NZ]	2.9	Asp 124 [O]
Asp 131 [OD1]	3.2	Trp 113 [NE1]
Asp 131 [OD1]	3.4	Arg 116 [NH1]

Supplementary Table S2. Table summarizing interacting residues of the interface between chain A and chain C.

Primer Name	Sequence
LC/A1 5’	CTC GTC GGG ATC CGC TGG AAG TGC TGT TTC AGG GCC CGT TTG TGA ACA AAC AGT TCA AC
LC/A1 3’	CAG GTC CTC GAG TTA TTA TTT GTT GTA GCC TTT GTC CAG AC
LC/A3 5’	CTC GTC GGG ATC CGC TGG AAG TGC TGT TTC AGG GCC CGT TTG TGA ACA AAC AGT TCA ATT ATC
LC/A3 3’	CAG GTC CTC GAG TTA TTA TTT GTT ATA GCC CTC ATC CAG AC
LC/A1 E171D 5’	GCT TTG GCC ACG ATG TTC TGA ATC TGA CCC GTA ATG G
LC/A1 E171D 3’	GTC AGA TTC AGA ACA TCG TGG CCA AAG CTT TTA CAT TC
LC/A3 D171E 5’	GCT TTG GCC ACG AAG TGT TTA ATC TGA CCC GTA ATG G
LC/A3 D171E 3’	GTC AGA TTA AAC ACT TCG TGG CCA AAG CTT TTA CAT TC
Avi tag-LC/A 5’ (LC/A1 and LC/A3)	CTC GTC GGA TCC GGC CTG AAC GAT ATT TTT GAA GCG CAG AAA ATT GAA TGG CAT GAA GGT TCA ATG CCG TTT GTG AAC AAA CAG TTC
DARPin-F5 5'	CTC GTC GGA TCC GGC CTG GTG CCG CGT GGT AGC GAC CTG GGT AAG AAA CTG CTG GAA GC
DARPin-F5 3'	CTC GTC AAG CTT ATT AAT TAA GTT TAG CAG CTT TCT GCA GAA CTT CAG C

Supplementary Table S3. Table showing sequences of oligonucleotides.
