# Supplementary material for: A DARPin promotes faster onset of botulinum neurotoxin A1 action
Source: Nat Commun. 2023 Dec 18;14:8317. doi: 10.1038/s41467-023-44102-4 (PMC10728214; doi:10.1038/s41467-023-44102-4)
Supplement: Supplementary file 3 — Reporting Summary [file 41467_2023_44102_MOESM3_ESM.pdf]

nature portfolio

Corresponding author(s): Richard A. Kammerer

Last updated by author(s): Nov 20, 2023

Reporting Summary

Nature Portfolio wishes to improve the reproducibility of the work that we publish. This form provides structure for consistency and transparency in reporting. For further information on Nature Portfolio policies, see our Editorial Policies and the Editorial Policy Checklist.

Statistics

For all statistical analyses, confirm that the following items are present in the figure legend, table legend, main text, or Methods section.

n/a	Confirmed	
☐	☒	The exact sample size (*n*) for each experimental group/condition, given as a discrete number and unit of measurement
☐	☒	A statement on whether measurements were taken from distinct samples or whether the same sample was measured repeatedly
☒	☐	The statistical test(s) used AND whether they are one- or two-sided<br>*Only common tests should be described solely by name; describe more complex techniques in the Methods section.*
☒	☐	A description of all covariates tested
☒	☐	A description of any assumptions or corrections, such as tests of normality and adjustment for multiple comparisons
☐	☒	A full description of the statistical parameters including central tendency (e.g. means) or other basic estimates (e.g. regression coefficient) AND variation (e.g. standard deviation) or associated estimates of uncertainty (e.g. confidence intervals)
☒	☐	For null hypothesis testing, the test statistic (e.g. *F*, *t*, *r*) with confidence intervals, effect sizes, degrees of freedom and *P* value noted<br>*Give P values as exact values whenever suitable.*
☒	☐	For Bayesian analysis, information on the choice of priors and Markov chain Monte Carlo settings
☒	☐	For hierarchical and complex designs, identification of the appropriate level for tests and full reporting of outcomes
☒	☐	Estimates of effect sizes (e.g. Cohen's *d*, Pearson's *r*), indicating how they were calculated

Our web collection on statistics for biologists contains articles on many of the points above.

Software and code

Policy information about availability of computer code

Data collection	- Crystallographic data was collected at beamline PXIII (Swiss Light Source, Villigen, Switzerland) equipped with an EIGER 16M high-resolution detector (Dectris).<br>- SPR binding data were collected on ProteON XPR36 instrument (BioRad) and on OpenSPR instrument (Nicoya)<br>- MPN assay was recorded with i-WORX 118 system (Dover, NH, USA)
Data analysis	- Crystallographic data analysis (XDS, PHENIX, COOT, PyMOL 2.4.0)<br>- BiaEvaluation software (Version 4.1) was uded to fit models to the LC/A1 binding data<br>- Tracedrawer Software (Ridgeview Instruments AB) was used to fit models to the LC/A3 binding data<br>- i-WORX 118 system (Dover, NH, USA) interfaced via Labscribe software (iWorx Systems Inc., Dover, NH, USA) for recording and analysis of the MPN assay.<br>-GraphPad Prism 7.02 and Microsoft Excel 2021 were used to analyze data.

For manuscripts utilizing custom algorithms or software that are central to the research but not yet described in published literature, software must be made available to editors and reviewers. We strongly encourage code deposition in a community repository (e.g. GitHub). See the Nature Portfolio guidelines for submitting code & software for further information.

Data

Policy information about availability of data

All manuscripts must include a data availability statement. This statement should provide the following information, where applicable:

- Accession codes, unique identifiers, or web links for publicly available datasets
- A description of any restrictions on data availability
- For clinical datasets or third party data, please ensure that the statement adheres to our policy

All the data used in this paper are available from the corresponding author on reasonable request. Atomic coordinates for LC/A1-DARPin-F5 have been deposited in the Protein Data Bank database under accession number 8HKH (https://doi.org/10.2210/pdb8HKH/pdb).

Human research participants

Policy information about studies involving human research participants and Sex and Gender in Research.

Reporting on sex and gender	N/A
Population characteristics	N/A
Recruitment	N/A
Ethics oversight	N/A

Note that full information on the approval of the study protocol must also be provided in the manuscript.

Field-specific reporting

Please select the one below that is the best fit for your research. If you are not sure, read the appropriate sections before making your selection.

☒ Life sciences ☐ Behavioural & social sciences ☐ Ecological, evolutionary & environmental sciences

For a reference copy of the document with all sections, see nature.com/documents/nr-reporting-summary-flat.pdf

Life sciences study design

All studies must disclose on these points even when the disclosure is negative.

Sample size	Most of the experiments were repetead independently twice (or three) to verify reproducibility. For MPN Assay we have n=5, because of sample availbility.
Data exclusions	No data were excluded from the analysis.
Replication	All attempts at replication were successful. The experiments were independently replicated at least twice and repeated at least three times within each of the experimental runs.
Randomization	Randomization was not a relevant feature as we were applying a uniform set of biochemical techniques across a set of recombinant proteins.
Blinding	Blinding was not performed during data collection, because not relevant in our case. For every treated sample we had a non-treated sample as control. Further more, the experiments were performed and analysed from the same operator.

Reporting for specific materials, systems and methods

We require information from authors about some types of materials, experimental systems and methods used in many studies. Here, indicate whether each material, system or method listed is relevant to your study. If you are not sure if a list item applies to your research, read the appropriate section before selecting a response.

Materials & experimental systems

n/a	Involved in the study
[ ]	[x] Antibodies
[ ]	[x] Eukaryotic cell lines
[x]	[ ] Palaeontology and archaeology
[ ]	[x] Animals and other organisms
[x]	[ ] Clinical data
[x]	[ ] Dual use research of concern

Methods

n/a	Involved in the study
[x]	[ ] ChIP-seq
[x]	[ ] Flow cytometry
[x]	[ ] MRI-based neuroimaging

Antibodies

Antibodies used	Anti-SNAP-25 (1:5,000, SMI81, Abcam, ab24737); anti-VAMP-2 (1:2,000, Synaptic System, 104 211); anti-SNAP-25 BoNT/A-cleaved was homemade and used as previously described in Duregotti et al., 2015; anti-Syntaxin-1A/1B was homemade and used as previously described in Zanetti et al., 2017.
Validation	The commercial antibodies used in the study have been indicated with supplier name and catalog number, the validation statements can be found on the manufacturer's website.<br>Instead characterization of the homemade antibodies have been previously reported (Antonucci et al., 2008; Antonucci et al., 2009; Pirazzini et al., 2014; Azarnia Tehran et al., 2015).

Eukaryotic cell lines

Policy information about cell lines and Sex and Gender in Research

Cell line source(s)	Cell lines: Cultured Cerebellar Granule Neurons (CGN) used in this study were obtained from a primary culture of cerebellar granule cells from post-natal rodent cerebellum.
Authentication	None of the cell lines used were authenticated.
Mycoplasma contamination	Cell lines were not tested for mycoplasma contamination.
Commonly misidentified lines (See ICLAC register)	No commonly misidentified cell lines were used in the study.

Animals and other research organisms

Policy information about studies involving animals; ARRIVE guidelines recommended for reporting animal research, and Sex and Gender in Research

Laboratory animals	Swiss-Webster adult male CD1 mice (20–24 g, 3 months old) were housed under controlled light/dark conditions, and food and water were provided ad libitum. The CD-1 mice were used to obtain the diaphragmatic muscles.
Wild animals	No wild animals were used.
Reporting on sex	Sex was not considered in the study design because no relevent for the experiment.
Field-collected samples	No field-collected samples were used.
Ethics oversight	The experiments were performed in accordance with the Italian laws and policies (D.L. no 26 14th March 2014), with the guidelines established by the European Community Council Directive no 2010/63/UE and approved by the Italian Ministry of Health and veterinary services of the University of Padova (O.P.B.A.-Organismo Preposto al Benessere degli Animali) (protocol 359/2015).

Note that full information on the approval of the study protocol must also be provided in the manuscript.
